# Breakdown of the Blood-Brain Barrier during Tick-Borne Encephalitis in Mice Is Not Dependent on CD8^+^ T-Cells

**DOI:** 10.1371/journal.pone.0020472

**Published:** 2011-05-23

**Authors:** Daniel Růžek, Jiří Salát, Sunit K. Singh, Jan Kopecký

**Affiliations:** 1 Institute of Parasitology, Biology Centre of the Czech Academy of Sciences, České Budějovice, Czech Republic; 2 Veterinary Research Institute, Brno, Czech Republic; 3 Section of Infectious Diseases and Immunobiology, Centre of Cellular and Molecular Biology, Hyderabad, India; Veterinary Laboratories Agency, United Kingdom

## Abstract

Tick-borne encephalitis (TBE) virus causes severe encephalitis with serious sequelae in humans. The disease is characterized by fever and debilitating encephalitis that can progress to chronic illness or fatal infection. In this study, changes in permeability of the blood-brain barrier (BBB) in two susceptible animal models (BALB/c, and C57Bl/6 mice) infected with TBE virus were investigated at various days after infection by measuring fluorescence in brain homogenates after intraperitoneal injection of sodium fluorescein, a compound that is normally excluded from the central nervous system. We demonstrate here that TBE virus infection, in addition to causing fatal encephalitis in mice, induces considerable breakdown of the BBB. The permeability of the BBB increased at later stages of TBE infection when high virus load was present in the brain (i.e., BBB breakdown was not necessary for TBE virus entry into the brain), and at the onset of the first severe clinical symptoms of the disease, which included neurological signs associated with sharp declines in body weight and temperature. The increased BBB permeability was in association with dramatic upregulation of proinflammatory cytokine/chemokine mRNA expression in the brain. Breakdown of the BBB was also observed in mice deficient in CD8^+^ T-cells, indicating that these cells are not necessary for the increase in BBB permeability that occurs during TBE. These novel findings are highly relevant to the development of future therapies designed to control this important human infectious disease.

## Introduction

Diseases that affect the central nervous system (CNS) also frequently alter the function of the blood-brain barrier (BBB) [1]–[5]. The BBB is formed by epithelial-like, high-resistance, tight junctions within the endothelium in capillaries perfusing the vertebrate brain, and represents a physical barrier between the bloodstream and the brain parenchyma. Integrity of the BBB is important for regulating the flow of nutrients from the blood to the brain and restricting access of toxins, pathogens, and other substances that are harmful to the CNS. Moreover, the BBB also restricts the access of inflammatory cell to the CNS, which protects irreplaceable cells (such as neurons) from damage [5].

Studies in models of CNS autoimmunity and virus-induced neuroinflammation have provided evidence linking the development of a CNS inflammatory response with enhanced BBB permeability. For example, an increase in BBB permeability has been associated with CNS inflammation in multiple sclerosis [6], experimental allergic encephalomyelitis [7], Borna disease [8], lymphocytic choriomeningitis [1], mouse adenovirus type 1 encephalomyelitis [5], rabies virus infection [9], West Nile virus (WNV) encephalitis [10], Japanese encephalitis virus (JEV) infection [11], Semliki Forest encephalitis, Banzi virus encephalitis [4], and in neuroAIDS [12]. It may be expected that enhanced BBB permeability is required to allow circulating immune cells contact with CNS tissue-derived chemokines, as well as to allow immune effectors to infiltrate CNS tissues. However, the exact mechanism of BBB breakdown and its role in the disease progression of viral encephalitis remain largely unknown [4].

Tick-borne encephalitis (TBE) is a human viral infectious disease involving the CNS, and is caused by the TBE virus (TBEV; family *Flaviviridae*, genus Flavivirus). The disease most often manifests as meningitis, encephalitis, or meningoencephalitis. More than 13,000 human clinical cases of TBE are reported across Europe and Asia annually. The number of TBE cases in all endemic regions of Europe has increased dramatically in the last 30 years; risk areas have spread and new foci have been discovered [13]. Therefore, TBE has become a growing public health problem.

Although TBEV is a neurotropic virus, the mechanisms underlying how TBEV gains access to the CNS are not completely understood [14], [15]. There are several hypothetical routes for the entry of TBEV into the CNS, including direct crossing of the BBB [14]. The mode by which TBEV crosses the BBB is unknown; however, several different hypotheses exist. These include (i) cytokine-mediated BBB breakdown, (ii) “Trojan horse” theory, and (iii) virus entry into the vascular endothelial cells of brain capillaries, transcytosis, and the release of virus into the brain parenchyma [14], [15]. In the case of cytokine-mediated CNS entry, cytokines such as tumor necrosis factor alpha (TNF-α) and interleukin 6 (IL-6) increase BBB permeability, which in turn can lead to the passage of virus into the CNS [10]. In human TBE patients, increased serum levels of TNF-α were observed during the first week of hospitalization [16]. Moreover, experiments on rats and mice showed that IL-6 causes disruption of the BBB [17]. The so-called “Trojan horse” mechanism is based on the migration of TBEV-infected cells of the immune system (e.g., dendritic cells, neutrophils, monocytes, macrophages, T-cells) through the BBB into the CNS, and initialization of TBEV infection in the CNS tissue [14]. Infection and replication of TBEV in endothelial cells or the choroid plexus and budding of the virus on the parenchymal side represent another possible explanation. However, in fatal TBE cases, TBEV antigens were not detectable in endothelial cells from brain sections. In patients with a severe form of TBE, neurospecific proteins such as α-1 brain globulin or neuron-specific enolase were elevated in serum, indicating BBB breakdown [18], [19]. However, despite this finding, dysfunction of the BBB has not been extensively studied in TBE so far.

In this study, we focused on BBB permeability during TBEV infection in two strains of mice. We reveal that infection of mice with TBEV increases BBB permeability and that BBB breakdown is largely independent of the presence of CD8^+^ T-cells. Moreover, we clearly demonstrate that the degree of BBB permeability corresponds with proinflammatory cytokine/chemokine expression in the brain, and consequently with disease progression.

## Results

**Figure 1:**
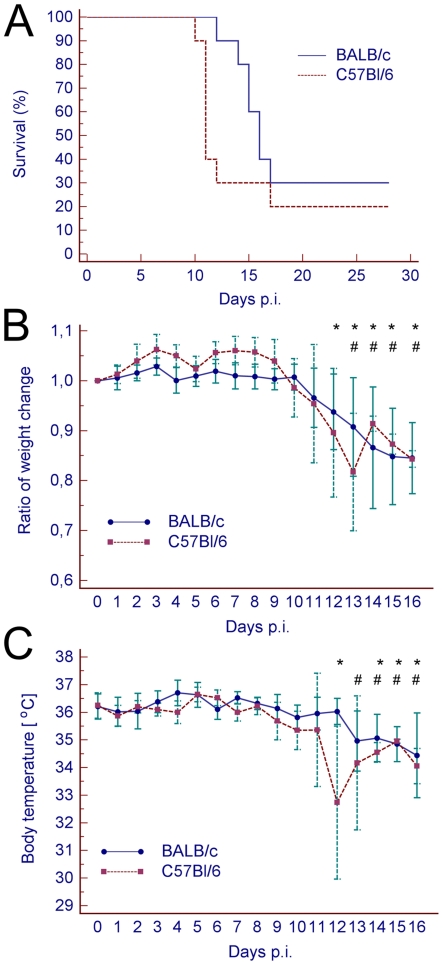
The effects of TBEV infection on BALB/c and C57Bl/6 mice. (A) Survival of BALB/c (n = 10) and C57Bl/6 (n = 10) mice subcutaneously inoculated with TBEV (100 pfu). (B) Body weight change ratio (as compared to the day 0) and (C) daily temperature fluctuation in TBEV-infected BALB/c (n = 10) and C57Bl/6 (n = 10) mice. For each time point the measured values are the average of the surviving mice. Error bars represent standard deviations. Statistically significant (p<0.05) decreases in body weight/temperature at different time points versus the beginning of the experiment are marked by an asterisk (C57Bl/6) or hash (BALB/c).

Groups of BALB/c and C57Bl/6 mice were subcutaneously inoculated with 100 pfu of the TBEV strain Neudoerfl/IRE. These two mouse strains with predominantly different Th immune responses (Th1 and Th2, for C57Bl/6 and BALB/c, respectively) were selected because of their different susceptibility to some viral neuroinfections [20], including Far Eastern TBEV [21]. Moreover, a previous report demonstrated a difference in BBB permeability between these strains (i.e., elevated permeability in C57Bl/6, but not in BALB/c mice after WNV inoculation) [22]. Survival rate and average survival times of TBEV-infected mice were compared. Following the same TBEV challenge, observed mortality was 70% in BALB/c mice and 80% in C57Bl/6 mice (Figure 1A). Although BALB/c mice exhibited a slightly longer mean survival time (19.0±6.0 days) in comparison with C57Bl/6 mice (15.0±6.7 days), the difference was not statistically significant (p>0.05).

Mouse body weight and temperature were determined prior to infection, and then daily throughout the course of the experiment, to determine relation of body weight and temperature (as indicators of the disease progression) with changes in BBB permeability. Body weight increased slightly during the first three days of infection. At days 4 and 5 post-inoculation (p.i.), there was a decrease in body weight in both mouse strains. This decrease was not statistically significant, but it was reproducible. After day 5 p.i., body weight again increased slightly. However, both strains of mice showed a sharp decrease in body weight beginning on day 10 p.i. in both mouse strains. A statistically significant decrease in body weight (p<0.05) was observed from days 12 and 13 p.i. in C57Bl/6 and BALB/c mice, respectively. At the termination of the experiment (on day 16 p.i.), most animals weighed approximately 85% of their original body weight (Figure 1B).

Similarly, the body temperature of infected mice decreased significantly (p<0.05) starting from day 12 and 13 p.i. in C57Bl/6 and BALB/c mice, respectively (Figure 1C). Mice with abnormally low body temperature (less than 32°C) were euthanized. None of the mice developed fever during the experiment.

Decreases in body weight and temperature corresponded with an appearance of clinical symptoms of the disease. All TBEV-inoculated mice remained asymptomatic for several days. Starting on day 10–13 p.i., most mice developed bristle coat, hunched back, and lethargic movement. Later, the disease symptoms progressed to lethargy with paralysis.

**Figure 2:**
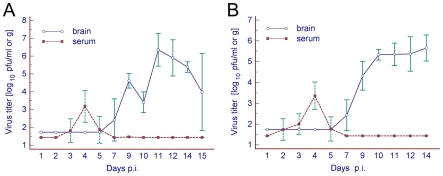
TBEV burden in serum and brain. (A) BALB/c and (B) C57Bl/6 mice were subcutaneously inoculated with TBEV (100 pfu). Brain and serum specimens were titrated individually by plaque assay on porcine kidney stable cell monolayers. For each time point the titers are the average of three mice. Error bars represent standard deviations. Detection limit for serum  = 1.4 log_10_ pfu/ml; brain  = 1.7 log_10_ pfu/g.

Serum samples and brains were harvested for titration at various time points during the experiment. The virus titers in the individual samples were determined by plaque assay on PS cells. Peak viremia was detected in both mouse strains on day 4 p.i. and reached approximately 3–3.5 log_10_ pfu/ml. From day 7 p.i. to the termination of the experiment, no virus was detected in any serum sample. Virus in the brain was first detected on day 7 p.i. in both mouse strains. Subsequently, the virus titer in brains of both mouse strains increased rapidly and reached a maximum of approximately 6–6.5 log_10_ pfu/g (Figure 2).

**Figure 3:**
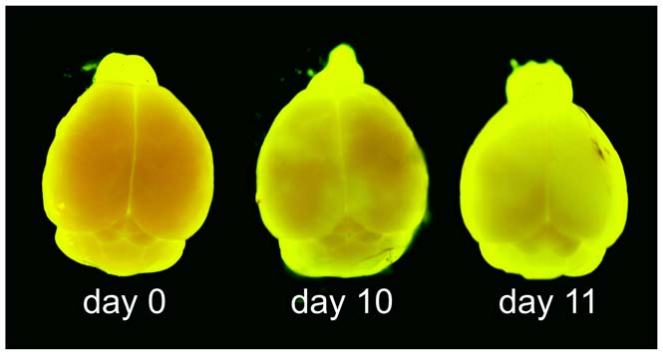
BBB permeability changes in the brains of TBEV-infected C57Bl/6 mice. Brains were removed from sodium fluorescein-treated mice, either uninfected (day 0) or TBEV-infected (days 10 and 11 after inoculation). The brains were photographed under UV illumination.

**Figure 4:**
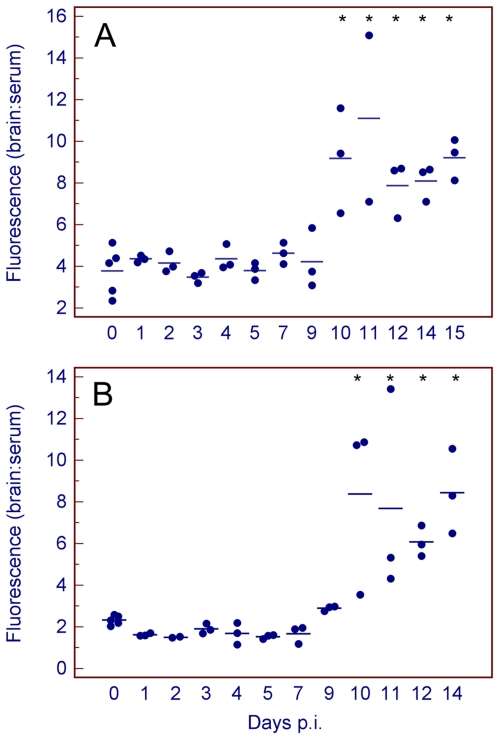
Measurement of BBB permeability using sodium fluorescein in (A) BALB/c and (B) C57Bl/6 mice. All mice were inoculated subcutaneously with TBEV (100 pfu). BBB permeability is expressed as brain:serum ratio (%). Thirty minutes after intraperitoneal injection of sodium fluorescein, the sera and brains were processed as described in the Material and Methods section and the fluorescence was measured. Asterisks indicate statistically significant increases in brain:serum fluorescence in infected versus uninfected mice (p<0.05).

TBEV-infected mice were assayed for BBB integrity at different time points using sodium fluorescein, a compound that is normally excluded from the CNS, and compared with mock-infected individuals. As measured by the leakage of sodium fluorescein from the circulation into the CNS tissues, BBB permeability began rising at day 10 p.i. in both mouse strains (i.e., when high virus titer was present in the brain, at the appearance of severe clinical signs of infection). Evidence of sodium fluorescein accumulation in the brain parenchyma was also observed when intact brains from infected mice were examined under ultraviolet (UV) light (Figure 3). The values of brain fluorescence in BALB/c and C57Bl/6 mice reached similar levels; however, the basal fluorescence was slightly higher in mock-infected BALB/c mouse brains than in brains from C57Bl/6 mice (2–4%) (Figure 4A). In C57Bl/6 mice, the brain fluorescence reached medium levels of 6–8% of serum fluorescence at later stages of the infection, which is more than 3- to 4-fold higher than the 2% noted in mock-infected mice (Figure 4B).

**Figure 5:**
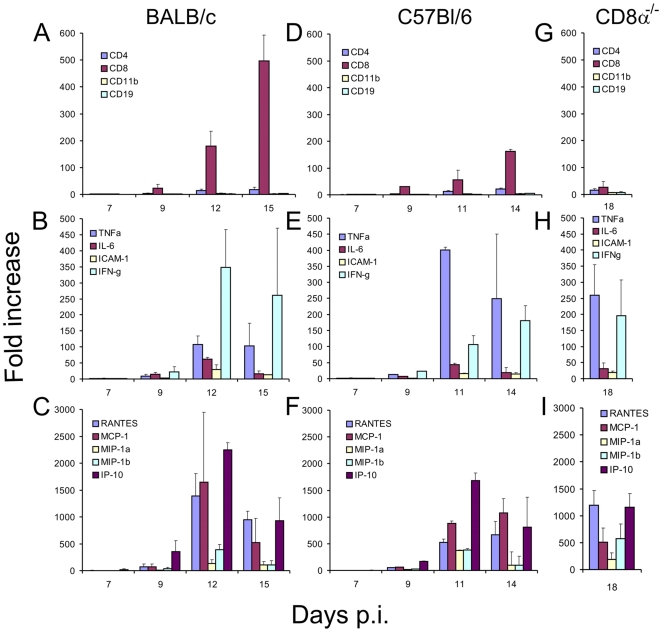
Immune/inflammatory cell accumulation and differential upregulation of cytokine/chemokine gene expression in the brain after TBEV infection. (A–C) BALB/c, (D–F) C57Bl/6, and (G–I) CD8α^−/−^ mice were infected subcutaneously with TBEV (100 pfu). CD8α^−/−^ mice were examined at the terminal stage (i.e. phase of neurological signs) of TBEV infection. Data are expressed as the mean + standard deviation of the fold increase of expression after normalization to the housekeeping gene (mouse beta actin).

The levels of mRNAs specific for the chemokines Regulated upon Activation, Normal T-cell Expressed, and Secreted (RANTES); C-C motif ligand 5 (CCL5); monocyte chemotactic protein-1 (MCP-1, also known as CCL2); macrophage inflammatory protein-1α (MIP-1α, also known as CCL3); MIP-1β (CCL4); and interferon-γ-inducible protein-10 (IP-10) were determined in the brain tissue of TBEV-infected BALB/c and C57Bl/6 mice at various times p.i. and compared with levels in the brains of uninfected mice (Figure 5). At day 9 p.i., before the BBB becomes significantly impaired, RANTES, MCP-1, IP-10, and to a lesser extent MIP-1β were slightly, but significantly (p<0.05) elevated in both infected mouse strains. On day 12 (BALB/c) or 11 p.i. (C57Bl/6) (i.e., during significant BBB disruption) expression of all chemokines was dramatically elevated. The highest increases were observed in IP-10, MCP-1, and RANTES mRNA levels. At the termination of the experiment, the increases of IP-10, MIP-1α, and MIP-1β mRNAs were lower than on days 11 or 12 p.i. in both mouse strains (Figure 5).

Similarly, the mRNA expression of proinflammatory cytokines TNF-α, IL-6, and IFN-γ became significantly (p<0.05) upregulated on day 9 p.i. in both mouse strains (i.e., one day before BBB permeability increased). Subsequently, the expression dramatically increased and remained at high levels until the termination of the experiment. TNF-α and IL-6 expression are of particular interest, because of their known contribution to increasing BBB permeability. Inter-cellular adhesion molecule-1 (ICAM-1) mRNA levels became increased on day 11 and 14 (C57Bl/6), or 12 and 15 (BALB/c) p.i. Interestingly, infected BALB/c mice exhibited a stronger IFN-γ response than C57Bl/6 mice, while C57Bl/6 mice had greater upregulation of TNF-α mRNA expression (Figure 5).

The changes in mRNA expression of cell surface markers of B-cells (CD19), T helper cells (CD4), cytotoxic T cells (CD8β), and macrophage/monocyte/granulocyte/NK cells (CD11b) in brain tissue after TBEV infection were determined. CD8β, CD4, CD11b, and CD19 mRNA in brain tissue became significantly higher (p<0.05) than those of normal uninfected mice by day 9 p.i., and continued to accumulate until the termination of the experiment. The increase was the highest in case of CD8β mRNA, much lower in case of CD4, and the lowest in case of CD11B and CD19 mRNA. The kinetics were analogous in infected BALB/c and C57Bl/6 mice (Figure 5).

**Figure 6:**
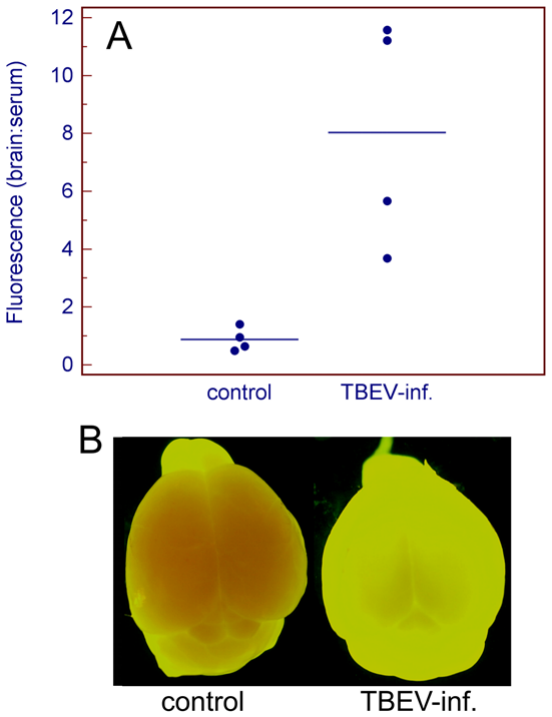
Measurement of BBB permeability using sodium fluorescein in CD8α^−/−^ mice. Mice were inoculated subcutaneously with TBEV (100 pfu). BBB permeability is expressed as brain:serum ratio (%). (A) Thirty minutes after intraperitoneal injection with sodium fluorescein, the sera and brains were processed as described in the Material and Methods section and the fluorescence was measured. Each line segment represents the mean of four samples (points). The increase in brain:serum fluorescence in infected versus uninfected mice was statistically significant (p<0.05). (B) Sodium fluorescein accumulation in the brains of CD8α^−/−^ before infection and at the terminal phase (i.e. phase with neurological signs) of TBE. The brains were photographed under UV illumination.

To determine whether TBEV-induced breakdown of the BBB is mediated by the migration of CD8^+^ T-cells into the brain parenchyma, an animal model lacking CD8^+^ T-cells (CD8α^−/−^ mice) was employed. CD8α^−/−^ mice exhibited slower development of the disease in comparison with the parent C57Bl/6 mice. The neurological signs appeared in CD8α^−/−^ mice approximately on days 16–17 p.i. Viral loads in the brains of CD8α^−/−^ mice during the neurological stage of infection were slightly higher (6.7–7.7 log_10_ pfu/g) when compared to the brains of BALB/c and C57Bl/6 mice. All investigated infected mice showed breakdown of the BBB, as detected by an increase in brain sodium flourescein levels compared to mock-infected mice. Brain fluorescence reached medium levels of approximately 8% of serum fluorescence at later stages of the infection in CD8α^−/−^ mice, which is more than 5-fold higher than the 1-1.5% noted in mock-infected mice (Figure 6A). This difference was statistically significant (p<0.05). Clear evidence of sodium fluorescein accumulation in the brains of moribund CD8α^−/−^ mice was also observed when intact brains from infected and control mice were examined macroscopically under UV light (Figure 6B). Therefore, BBB breakdown occurs in both immunocompetent and CD8α^−/−^ mice, despite the lack of CD8^+^ T-cell mediated inflammation in CD8α^−/−^ mice.

Relative mRNA quantitation by real-time RT-PCR showed that the CD8α deficiency had no effect on cytokine or chemokine mRNA levels in the brain tissue of infected mice. At the terminal phase of infection (phase of neurological signs of the disease), the levels of all chemokines/cytokines tested were analogous in TBEV-infected CD8α^−/−^ mice and in C57Bl/6 mice on day 14 p.i. (Figure 5). Similarly, the levels of CD4, CD11b, and CD19 mRNA in the brains of infected CD8α^−/−^ mice reached levels analogous to those in the brains of infected C57Bl/6 mice. Interestingly, despite the deficiency in the CD8α chain in CD8α^−/−^ mice, we were able to detect an increase in CD8β mRNA production at later stages of infection versus the level in uninfected CD8α^−/−^ mice. However, this increase was only very slight in comparison with the remarkable increase observed in infected C57Bl/6 mice.

## Discussion

The BBB is more than just a barrier that separates the CNS from the blood. It is also a crucial regulatory interface through which nutrients, xenobiotics, cytokines, immune cells, and pathogens pass into and out of the CNS [12]. Brain homeostasis is critically dependent on the integrity of the BBB. Alteration of the BBB is a hallmark feature of many CNS infections and likely has important effects on disease progression [4]. Encephalitis associated with breakdown of the BBB can be caused by a number of encephalitic RNA and DNA viruses [23]. Within the family *Flaviviridae*, BBB breakdown was observed in WNV [10], JEV [11], and dengue [24] infections. We demonstrate here that TBEV infection, in addition to causing fatal encephalitis in susceptible mouse strains, also causes breakdown of the BBB. Permeability of the BBB began rising at the time of the appearance of severe clinical signs of infection, including neurological symptoms associated with sharp declines in body weight and temperature [21], and occurred at a time when high virus titer had been already present in the brain. No increase in BBB permeability was observed during the viremic phase of the infection. Therefore, TBEV is able to enter the brain through the intact BBB. Unlike WNV encephalitis [22], a similar pattern in BBB breakdown was observed in BALB/c and C57Bl/6 mice. In human patients with the severe form of TBE, BBB breakdown has been suggested based on the increased level of neurospecific proteins, such as α-1 brain globulin or neuron-specific enolase, in serum [18], [19]. However, our study is the first to demonstrate BBB breakdown during TBEV infection experimentally.

In the case of WNV encephalitis in mice, increased BBB permeability induced by TNF-α allows WNV to cross into the CNS [10]. BBB breakdown occurs at day three after infection, when viremia and WNV burden are high in the periphery [10], [25]. BBB breakdown in the context of WNV is dependent on the Toll-like receptor (TLR)-3, since TLR-3 deficient mice exhibit increased survival when compared with wild-type mice after infection. Moreover, CNS inflammatory cytokines, leukocytes, virus titers, and pathological changes were decreased in TLR-3–deficient mice [10]. However, these findings seemed to be specific to WNV only, since the deficiency in TLR-3 had no effect on the pathogenesis of four other viral infections (lymphocytic choriomeningitis virus, vesicular stomatitis virus, murine cytomegalovirus, and reovirus) [26]. Another study demonstrated that the increased BBB permeability in WNV-infected rodents is not a primary determinant for lethality [22]. Similarly to our study, an increase in BBB permeability in correlation with disease progression was observed in JEV-infected mice [11].

The increase of BBB permeability in TBEV-infected mice corresponded with excessive mRNA expression of several chemokines and cytokines in the brain parenchyma. Dramatic increases in the expression of cytokines/chemokines TNF-α, IL-6, IFN-γ, ICAM-1, RANTES, MCP-1, MIP-1α, MIP-1β, and IP-10 positively correlated with an increase in BBB permeability. C57Bl/6 mice exhibited a stronger TNF-α response than BALB/c mice, which is in agreement with the dominant, proinflammatory, Th1-type immune response of C57Bl/6 mice. On the other hand, BALB/c mice had higher IFN-γ expression, corresponding with a higher amount of CD8^+^ T-cells observed in the brain tissue of these mice. Expression of ICAM-1 (BALB/c and C57Bl/6), MIP-1α (BALB/c and C57Bl/6), and MIP-1β (C57Bl/6) became increased later (i.e., after the initiation of BBB breakdown). Similarly to our study, the extent of BBB permeability was in direct association with increased levels of cytokines, clinical sickness, and virus titer in the brains of JEV-infected mice [27]. Robust release of some of the investigated cytokines/chemokines in the brains of TBEV-infected mice has also been recently demonstrated by other authors [28], [29]. It is known that the overproduction of proinflammatory cytokines, including TNF-α and IL-6, enhances neuronal injury [30] and increases BBB permeability [31], [32]. MCP-1 is also known as an important stimulus capable of causing BBB breakdown [33], [34]. In human TBE patients, the highest levels of serum TNF-α and IL-6 are observed during the first week of hospitalization, and treatment reducing these cytokines leads to quicker improvement and faster recovery [16]. Similarly, increase of MCP-1 [35] and RANTES [36] in the cerebrospinal fluid of TBE patients was observed.

Under normal conditions, there are minimal numbers of lymphocytes detected within the CNS, a result of reduced penetration of lymphocytes across the BBB. However, during inflammatory conditions, there is a significant accumulation of lymphocytes in the brain parenchyma. It has been demonstrated in several brain diseases, such as multiple sclerosis, stroke, neuroAIDS, and Alzheimer's disease, that BBB breakdown is associated with leukocyte migration into the brain [37], [38]. Leukocyte recruitment can trigger signal transduction cascades leading to junctional disorganization and BBB breakdown [37]. Immune-mediated CNS vascular permeability is a likely contributor to pathology in infectious CNS disease, and leukocyte migration into the brain tissue often has devastating consequences [39], [40].

In this study, we have demonstrated that there is significant increase of cells expressing CD8β antigen in the brain parenchyma of both BALB/c and C57Bl/6 mice, starting on day 9 p.i. The amount of CD8β^+^ cells increased until the termination of the experiment. The increase of CD4^+^ T-cells was much lower, and CD11b- and CD19-expressing cells increased slightly but still significantly. These data indicate that CD8^+^ T-cells are the most prominent lymphocyte population crossing the BBB during TBE. Dual roles for these cells during TBE (protective and immunopathological) in mice have been demonstrated previously [41]. Moreover, data from histopathological investigation of brains from human fatal TBE cases support the hypothesis on the immunopathological role of cytotoxic T-cells in TBE [42], [43]. However, the role of CD8^+^ T-cells in BBB breakdown during flavivirus encephalitis remained largely unknown. It has been demonstrated that antigen-specific CD8^+^ T-cells initiate astrocyte activation, alteration of BBB tight junction proteins, and increased CNS vascular permeability. Perforin-deficient mice are resistant to tight junction alterations and CNS vascular permeability, indicating the involvement of perforin-dependent mechanism in CD8^+^ T-cell-mediated BBB breakdown [44]. In murine lymphocytic choriomeningitis, BBB breakdown is mediated by virus-specific CD8^+^ T-cells. These cells are mandatory for the increase in BBB permeability as well as for mortality [1]. On the contrary, breakdown of the BBB despite the absence of CD8^+^ T-cells has been observed in mouse adenovirus type-1 encephalomyelitis [5]. To directly test the role of CD8^+^ T-cells in the increase of BBB permeability during TBE, an experimental mouse model lacking a functional CD8α^+^ T-cell population was employed. Interestingly, significant BBB breakdown was observed at terminal phases of TBE in these CD8α^−/−^ mice. Therefore, TBEV-induced breakdown of the BBB is largely independent of the presence of CD8^+^ T-cells, although we cannot rule out possible contributions of these cells to the process. The mRNA expression of cytokines/chemokines in the brains of these TBEV-infected CD8α^−/−^ mice exhibited similarly increased levels as in TBEV-infected C57Bl/6 mice, indicating that even this phenomenon was not significantly affected by the absence of CD8α^+^ T-cells. The same is true for the increase of mRNAs specific for CD4, CD11b, and CD19 antigens. The role of the other inflammatory cells in the development of BBB breakdown remains unknown.

The mode of TBEV entry into the brain is largely unknown. A variety of flaviviruses have been speculated to enter the CNS via the olfactory pathway [45], [46] or across cerebral capillary endothelial cells [11], [47]. In this study, we showed that TBEV entry into the brain is not dependent on the breakdown of the BBB, but that BBB breakdown is a consequence of virus infection of the brain. Experiments with an in vitro BBB model may provide more information on the direct interaction of TBEV with brain microvascular endothelial (BMVE) cells, the main component of the BBB. A study on WNV demonstrated that human BMVE cells are susceptible to WNV infection and that cell-free WNV can cross the BBB without compromising the integrity of the BBB. These data suggest that infection of BMVE cells can facilitate the entry of cell-free virus into the CNS without disturbing the BBB [48].

In summary, we demonstrate here that TBE is associated with dramatic BBB breakdown occurring at later stages of infection when high virus titer is present in the brain, and the severe clinical symptoms appear. BBB breakdown is not necessary for TBEV entry to the brain and is not dependent on the migration of CD8^+^ T-cells into the brain. It more likely represents a bystander effect of virus-induced cytokine/chemokine overproduction in the brain. With respect do difficulties of drug delivery thought intact BBB under normal conditions, these novel findings are highly relevant to the development of therapies designed to control this serious human infection.

## Materials and Methods

### Mice

BALB/c and C57Bl/6 mice were obtained from Charles River Laboratories (Sulzfeld, Germany). CD8α-knockout mice of the C57Bl/6 background (CD8α^−/−^; strain B6.129S2-Cd8atm1Mac) were obtained from The Jackson Laboratory (BarHarbor, ME, USA). Sterilized pellet diet and water were supplied ad libitum. In all experiments, female mice aged 7–9 weeks were used. The mice were housed in plastic cages with wood-chip bedding, situated in a specific pathogen-free room with a constant temperature of 22°C and a relative humidity of 65%.

### Virus infection

The mice were infected subcutaneously with 100 pfu of TBEV, strain Neudoerfl/IRE. This strain was passaged three times in suckling mice and once in *Ixodes ricinus* cells (cell line IRE/CTVM19 [49]). In comparison to mice infected with highly passaged TBEV strains, Neudoerfl/IRE-infected mice exhibit longer mean survival time and increased survival. The parental strain, Neudoerfl, was originally isolated from the tick *I. ricinus* in Austria in 1971. The virus has been extensively characterized, including its complete genome sequence (GenBank accession number U17495), and the determination of the three-dimensional structure of its envelope protein E [50].

The body weight and temperature of infected mice were measured daily. Rectal temperature was determined using the Rodent Thermometer BIO-TK9882 (Bioseb).

### BBB permeability assay

Sodium fluorescein (Sigma-Aldrich), a low molecular mass molecule (376 Da), was used to detect fluid shifts between the circulation and CNS, which occur when BBB permeability becomes enhanced [9]. Mice were injected with 10 mg sodium fluorescein in 0.1 ml sterile saline i.p. Thirty minutes later, animals were anesthesized with ketamine-HCl (100–200 mg/kg), cardiac blood was collected, and mice were perfused with PBS to remove blood from the intravascular compartment. Brains were removed, individually weighed, and stored at −70°C until processing. Homogenization of brain tissues was performed in 1 ml sterile PBS using TissueLyser II (Qiagen). The homogenate was clarified by centrifugation at 14000× g for 10 min, at 4°C. Protein was precipitated with trichloroacetic acid (TCA) to remove potential background fluorescence. The supernatant was diluted 1∶10 in 15% TCA. Serum samples were initially diluted 1∶10 in PBS and subsequently 1∶10 in 15% TCA. All samples were incubated at 4°C overnight. Samples were then centrifuged at 14000× g for 10 min, 4°C, and the supernatant was diluted 1∶1 in borate buffer (0.05 M, pH 10). The amount of fluorescein in each sample was determined using standards ranging from 125 to 3000 µg in 7.5% TCA and 0.025 borate buffer on an Infinite M200 fluorometer (Tecan) using an excitation wavelength of 480 nm, and fluorescence was read at 538 nm.

The uptake ratio was expressed as the ratio of the amount of sodium fluorescein measured in the brain to the amount measured in serum.

To visualize sodium fluorescein accumulation in different regions of the CNS, the brain was exposed to UV light using the Visi-Blue UV Transilluminator (UVP Laboratory Products), and photographed with an Olympus X-710 digital camera.

### Plaque assay

The virus titers in brain homogenates and sera of infected mice were estimated by plaque assay on porcine kidney stable cell monolayers under a carboxymethyl-cellulose overlay, as described previously [51]. Infectivity was expressed in plaque-forming units (pfu) per ml of serum or g brain tissue.

### Real-time quantitative RT-PCR

Brain tissue pellets prepared as described above were used to quantify the expression levels of specific mRNAs in the CNS. RNA was isolated from the pellet using the RNeasy Mini Kit (Qiagen), according to the recommendations of the manufacturer. cDNA was synthesized using a High Capacity RNA-to-cDNA Kit (Applied Biosystems), according to the manufacturer's protocol. The synthesized cDNAs were used as templates for real-time PCR. Real-time quantitative PCR was performed using pre-developed TaqMan® Assay Reagents (assay IDs: Mm00443258_m1, TNF-α; Mm00446190_m1, IL-6; Mm00516023_m1, ICAM-1; Mm01168134_m1, INF-γ; Mm00445235_m1, IP-10; Mm00441242_m1, MCP-1; Mm00441258_m1, MIP-1α; Mm00443111_m1, MIP-1β; Mm01302428_m1, RANTES; Mm00442754_m1, CD4; Mm00438116_m1, CD8β1; Mm00434455_m1, CD11b; Mm00515420_m1, CD19; Nm_007393.1, mouse beta actin; Applied Biosystems) and TaqMan® Gene Expression Master Mix (Applied Biosystems) on a Rotor Gene-3000 (Corbett Research). Mouse beta actin was used as a housekeeping gene. The amplification conditions were as follows: initially, 2 minutes at 50°C (to allow UNG to destroy any contaminating templates); 10 minutes at 95°C (to denature UNG and activate the enzymes); 40 cycles of denaturation at 95°C for 15 seconds; and annealing/extension at 60°C for 1 minute.

To calculate the fold change in gene expression, the CT of the housekeeping gene was subtracted from the CT of the target gene to yield the ΔCT. Change in expression of the normalized target gene was expressed as 2^-ΔΔCT^ where ΔΔCT = ΔCT sample-ΔCT control, as described previously [52].

### Statistics

The survival of mice was analyzed using Kaplan-Meier survival analysis. All other data were analyzed by one-way ANOVA, followed by the Student-Newman-Keuls test for all pairwise comparisons. Prior to ANOVA, Levene's Test for Equality of Variances was performed. All statistical analyses were performed using MedCalc software, version 11.2.1.0.

### Ethics Statement

This study was carried out in strict accordance with the Czech national law and guidelines on the use of experimental animals and protection of animals against cruelty (the Animal Welfare Act Number 246/1992 Coll.). The protocol was approved by the Committee on the Ethics of Animal Experiments of the Institute of Parasitology and of the Departmental Expert Committee for the Approval of Projects of Experiments on Animals of the Academy of Sciences of the Czech Republic (Permit Number:152/2009).
